# Supplementary material for: Exploration of Somatostatin Binding Mechanism to Somatostatin Receptor Subtype 4
Source: Int J Mol Sci. 2022 Jun 21;23(13):6878. doi: 10.3390/ijms23136878 (PMC9266823; doi:10.3390/ijms23136878)
Supplement: Supplementary file 1 [file ijms-23-06878-s001.zip › ijms-1763091-Supplementary.pdf]

Supplementary Information

Exploration of somatostatin binding mechanism to somatostatin receptor subtype 4

Rita Börzsei[1,2], Balázs Zoltán Zsidó[1,2], Mónika Bálint[1,2], Zsuzsanna Helyes[1,2,3,4], Erika Pintér[1,2,3,4], Csaba Hetényi[1,2]

[1]*Department of Pharmacology and Pharmacotherapy, Medical School, University of Pécs, Hungary.*

[2]*János Szentágothai Research Centre & Centre for Neuroscience, University of Pécs, Hungary.*

[3]*Algonist Gmbh, Wien, Austria*

[4]*PharmInVivo Ltd. Hungary*

.

Table of Contents

Table S1 Abridged results of BLAST search showing the first four matches 2

Table S2 RMSD matrix of the best homology models derived from different templates 2

Table S3 The lowest interaction energy ligand copies in each wrapping cycle 2

Table S4 Target residues in 3.5 Å distance from SST in the external binding mode 3

Table S5 Interacting target residues in 3.5 Å distance from SS14 in the internal binding cleft 3

Table S6 DOPE score of homology models 3

Table S7 Lennard-Jones intermolecular interaction between SSTR4 and full SST ligand for external binding cleft 4

Table S8 The d_{SB} (Å) in homology models of internal binding position 4

Figure S1 The internal and the alternative mode of SST on SSTR4 compared with that of SSTR2 5

Figure S2 Per-residue energy analysis of the alternative SST:SSTR4 complex 6

Video S1 Binding mechanism of SST on SSTR4 6

Supporting Tables

Table S1 Abridged results of BLAST search showing the first four matches.

PDB code	Name of the structure	Max score	Total score	Query score	E value	Per. Ident
4n6h_A	Structure of the human delta opioid 7TM receptor (Homo sapiens)	242	242	77%	5 e-76	44.98%
4rwa_A	Synchrotron structure of the human delta opioid receptor in complex with a bifunctional peptide (Homo sapiens)	240	240	73%	2 e-75	46.74%
6dde_R	Mu Opioid Receptor-Gi Protein Complex (Mus musculus)	238	238	73%	4e-75	40.55%
5c1m_A	Crystal structure of active mu-opioid receptor bound to the agonist BU72 (Mus musculus)	235	235	72%	6e-75	40.83%

Table S2 RMSD matrix of the best homology models derived from different templates.

Name of the template sturcture	β_2-receptor (3p0g)	δ-opioid receptor (4n6h)	μ-opioid receptor (5c1m)
β_2-receptor (3p0g)	0	2.015	2.029
δ-opioid receptor (4n6h)	2.015	0	1.611
μ-opioid receptor (5c1m)	2.029	1.611	0

Table S3 The lowest interaction energy ligand copies in each wrapping cycle. The ligand copy with the best interaction energy is highlighted with grey and used in the next steps.

Docking cycle	Interaction energy of the ligand copy (kcal/mol)
1	- 10.23
2	- 8.25
3	- 4.71
4	- 1.92
5	- 0.74
6	- 2.08
7	0.28

Table S4 Target residues in 3.5 Å distance from SST in the external binding mode.

Target residues
R110
R188
P189
A190
R191
A197
C198
N199
S287
L288
D289

Table S5 Interacting target residues in 3.5 Å distance from SS14 in the internal binding cleft. Interacting target residues found for both internal binding mode models are highlighted in grey. Interacting target residues found for all internal and external binding modes are highlighted in blue.

Target residues	7th model (d_{SB}=3.1 Å)	9th model (d_{SB}=3.0 Å)
R110	x	
H111	x	
D126	x	x
A190	x	
V196	x	
A197	x	x
C198	x	x
N199	x	x
L200		x
T286		x
S287		x
D289	x	x
A290	x	
N293	x	
L297	x	
Y301	x	x

Table S6 DOPE score of homology models. The models with the best DOPE score are colored with grey.

Model number	Name of the template		
	β_2-receptor (3p0g)	μ-opioid receptor (5c1m)	δ-opioid receptor (4n6h)
1	-38064.36	-39480.23	-40644.86
2	-38377.65	-39293.33	-40807.62
3	-38223.52	-39453.68	-41277.31
4	-37831.44	-39540.48	-40887.89

5	-38198.88	-39586.63	-41458.39
6	-38289.45	-39958.14	-41770.93
7	-38619.66	-39316.14	-40288.13
8	-38644.30	-39433.41	-40802.17
9	-38398.09	-39591.16	-40880.65
10	-38302.61	-39407.04	-42014.23

Table S7 Lennard-Jones intermolecular interaction between SSTR4 and full SST ligand for external binding cleft. Model with the lowest interacting energy is highlighted in grey.

Model Number	5c1m-based superimposed model
1	-26.85
2	-50.55
3	-50.88
4	-54.51
5	-53.62
6	-50.15
7	-50.05
8	-39.34
9	-41.29
10	-40.87

Table S8 The d_{SB} (Å) in homology models of internal binding position. Models with the smallest distance are highlighted in grey.

Model number	No distance restraint during the model building	5 Å distance restraint on the amino N atom of K9:SST and the carboxylate C atom of D126:SSTR4 atom pair during the model building	6 Å distance restraint on the amino N atom of K9:SST and the carboxylate C atom of D126:SSTR4 during the model building
1	6.4	5.7	7.2
2	7.4	6.1	6.2
3	7.2	5.8	5.9
4	6.7	5.5	6.0
5	6.5	5.6	6.2
6	6.1	5.7	6.0
7	7.5	5.9	5.7
8	7.9	5.5	6.2
9	6.6	5.4	5.8
10	8.5	6.4	6.3

Supporting Figures

Figure S1 a) The internal binding mode of SST on SSTR4 and SSTR2 colored with green and salmon, respectively **b)** The alternative binding mode of SST (green) in SSTR4 with a ca. 180° flip compared with that (salmon) of SSTR2. In **a) and b)** receptors (grey) and ligands are in cartoon representation while W8K9:SST and D126:SSTRs are highlighted with sticks.

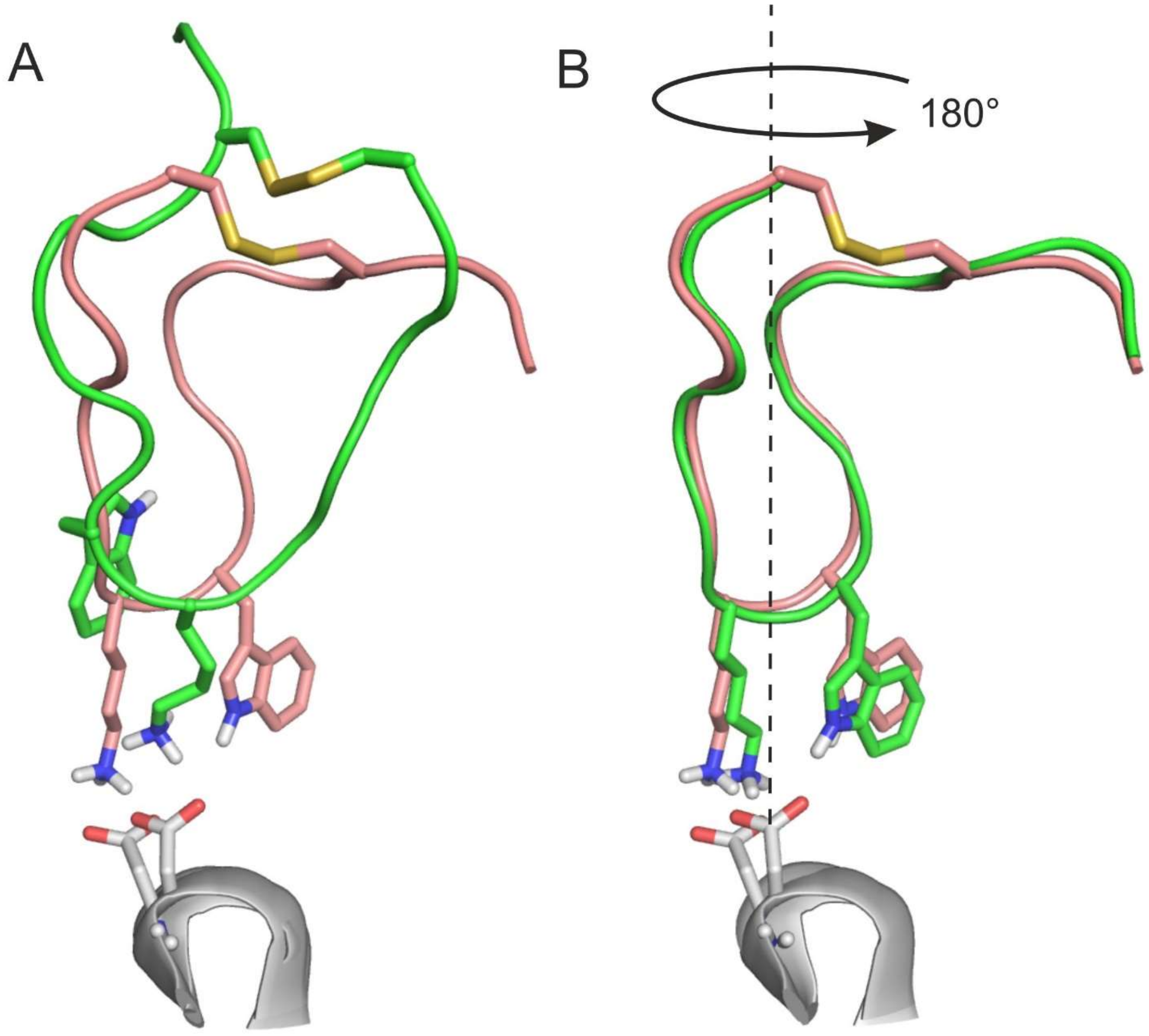

Figure S2 Per-residue energy analysis of the alternative SST:SSTR4 complex shown for both the receptor (SSTR4) and the alternative somatostatin (SST) sides.

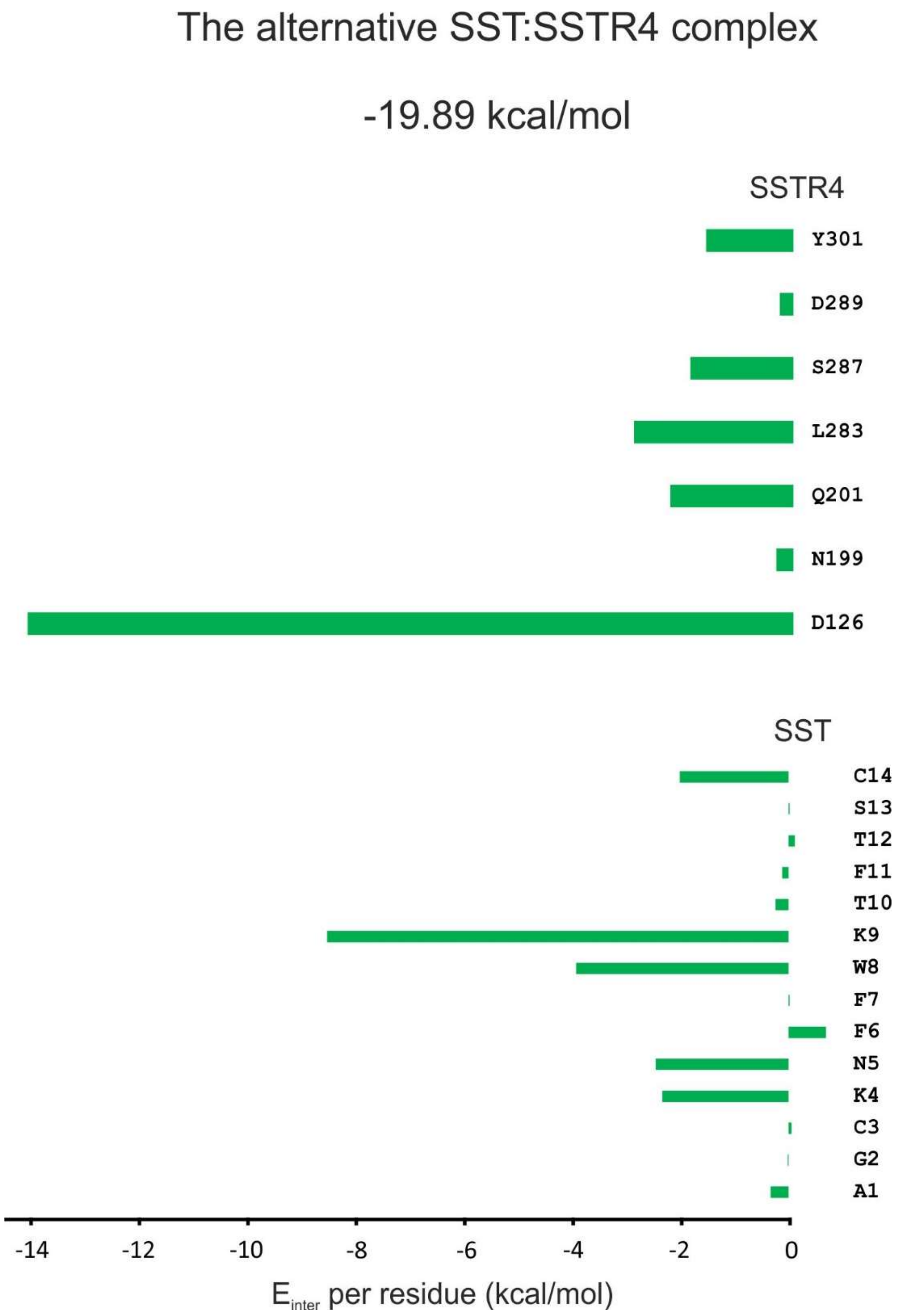

Video S1 Binding mechanism of SST (green, cartoon, K9 highlighted with sticks, all atom) including external, intermediate (~ 10 Å and ~ 5 Å), and internal binding modes on SSTR4 (grey, cartoon). D126 is highlighted with sticks.
